# Multiple kernels learning-based biological entity relationship extraction method

**DOI:** 10.1186/s13326-017-0138-9

**Published:** 2017-09-20

**Authors:** Xu Dongliang, Pan Jingchang, Wang Bailing

**Affiliations:** 1 School of Mechanical, Electrical and Information Engineering, ShanDong University, WenHua West Road, WeiHai, 264209 China; 2 School of Computer Science and Technology, Harbin Institute of Technology, WenHua West Road, WeiHai, 264209 China

**Keywords:** Tag-graph kernel, Entity relationship extraction, Multi-kernels learing

## Abstract

**Background:**

Automatic extracting protein entity interaction information from biomedical literature can help to build protein relation network and design new drugs. There are more than 20 million literature abstracts included in MEDLINE, which is the most authoritative textual database in the field of biomedicine, and follow an exponential growth over time. This frantic expansion of the biomedical literature can often be difficult to absorb or manually analyze. Thus efficient and automated search engines are necessary to efficiently explore the biomedical literature using text mining techniques.

**Results:**

The P, R, and F value of tag graph method in Aimed corpus are 50.82, 69.76, and 58.61%, respectively. The P, R, and F value of tag graph kernel method in other four evaluation corpuses are 2–5% higher than that of all-paths graph kernel. And The P, R and F value of feature kernel and tag graph kernel fuse methods is 53.43, 71.62 and 61.30%, respectively. The P, R and F value of feature kernel and tag graph kernel fuse methods is 55.47, 70.29 and 60.37%, respectively. It indicated that the performance of the two kinds of kernel fusion methods is better than that of simple kernel.

**Conclusion:**

In comparison with the all-paths graph kernel method, the tag graph kernel method is superior in terms of overall performance. Experiments show that the performance of the multi-kernels method is better than that of the three separate single-kernel method and the dual-mutually fused kernel method used hereof in five corpus sets.

## Background

There are more than 20 million literature abstracts included in MEDLINE, which is the most authoritative textual database in the field of biomedicine.The biomedical literature is difficult to detect manually because of growing number of papers. Thus biomedical entity relationship extraction is necessary to analysis biomedical literature.Biomedical entity relationship extraction is the extraction of inter-entity specific semantic relationships in text [1, 2]. Besides, it is benefit for semantic similarity [3], biological network construction [4, 5] and ontology term prediction [6, 7].

**Fig. 1:**
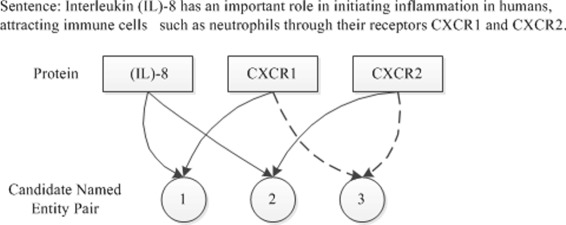
Sampling example of protein interaction (The PMID of the literature where the sentence is found is 23041326, and PMID refers to the retrieval number biological literature coded by PubMed)

In the biomedical texts, the entity relationships contain gene-disease association [8–10], drug-drug interaction [11–13], protein-protein interaction. Biomedical relation extraction aiming to automatically discover relations from these biomedical articles with high efficiency and accuracy, is becoming an increasingly well understood alternative to manual knowledge discovery. In this article, entity relationship extraction refers to the extraction of entity relationship that appears in the same sentence. Considering the extraction of protein interaction relationships as an example, as shown in Fig. 1. “Sentence” is a sentence comprising a natural language in the biological literature, i.e., an object to be extracted; “Protein” means a biological entity named protein, which is present in the sentence to be extracted, and three proteins coexist in the sentence in the figure, namely,“IL-8”,“CXCR1” and “CXCR2”, respectively. “Candidate Named Entity Pair” refers to the candidate relationship pairs comprising two proteins and three candidate entity relationship pairs contained in the sentence, as shown in the figure, two of which are correct protein relationship pairs. These relationship pairs are marked by two actual performance arrows in the figures. The entity relationship extraction is the accurate extraction of the two correct entity relationship pairs.

A knowledge network of biological entity can be predicted and established by extracting biological entity relationship [14]. A heavily studied area in biological text mining concerns the relationships known as protein-protein interactions (PPI). Massive PPI have accumulated continuously with the exponential growth of biomedical literature.

The remainder of the paper is organized as follows: Section II reviews the related work. Section III is overview of our approach, which contains introduction of our approach (A type of tag graph kernel method), Characteristics-based kernels, extension dependency path tree kernel and fused kernel method. In section IV, we construct an experiment to evaluate our approach and fused kernel method. Section V is our conclusion.

Biological entity relationship extraction methods can be categorized into three categories statistical machine learning method [15, 16], co-occurrence-based [17, 18] and pattern-based method [19, 20].

The co-occurrence-based method is a graphical representation of relationships between terms [21, 22]. Antono et al. [23] proposed new method known as WeMine-P2P based on WeMine Aligned Pattern Clustering algorithm which discovers and identifies the localized and co-occurring conserved patterns and regions allowing variable length and pattern variations.

Although the co-occurrence-based method is simple and easy to use, the hypothesis depended on by this method fails to completely reflect the actual situation of massive and complicated biological texts, therefore leading to a relatively poor accuracy. Therefore, the co-occurrence-based method is usually applied to the “crude extraction” stage, indicating that all candidate relationship pairs are extracted. The more accurate extraction of entity relationships requires fusing other information to filter the extracted candidate relationship pairs.

The patterns defined are used to match the labeled sequence in the pattern-based methods.The pattern-based method contains two methods: the method based on extraction-pattern [24] and the method based on template [25]. The extraction-pattern-based method summarizes entity relationship to obtain several extraction rules in the texts by using the natural language processing tool. The template-base method explores the entity relationships from the aspect of syntax or part of speech to summarize a series of templates by utilizing the natural language processing. Peng et al. [26] proposed a pattern-based biomedical relation extraction system with a new framework. There are three characteristics: 1) generating patterns by adjusting syntactic variations, 2) improving the coverage of patterns by using sentence simplification, 3) the referential relations can be identified. Some systems which are implemented by the pattern-base method depend on pre-defined patterns at the surface textual level [27–29].Other parsers are used with hand-crafted patterns [30–32].

Compared with the above two methods, machine learning-based approaches which are driven by data and set of annotated corpora are effective [33–36]. But the quality and the number of annotated corpora are significant effort to the performance of systems.

Machine learning-based approaches include the following two ways: supervised-machine-learning-based method [37] and semi-supervised-machine-learning-based method [38, 39]. Supervised machine learning methods have been employed with great success in PPI extraction. However, they usually require a large amount of annotated data for training which are expensive to obtain in practical applications. Kamada et al. [37] proposed a method to predict strengths of PPIs by employing protein domain information. Jiang et al. [38] proposed a multi-label correlated semi-supervised machine learning method. It can effectively solve the problem of labeled data by exploring the intrinsic relationship between related classes.

The semi-supervised-machine-learning-based method includes the method based on characteristic [40, 41] and the method based on kernel [42, 43].

In this paper, a type of tag graph kernel method for extracting protein relationship was proposed and combined with feature-based kernel and extension path graph kernel into a fused kernel learning method.

## Methods

In this article, the kernel method is used as a function to calculate the similarity between two objects. We used three kernels to calculate the inter-entity relationships from three aspects, which can avoid losing important features and strengthen similarity measurement.

### Characteristics-based kernels

Characteristic selection is the main work of using characteristic-based kernel function for extracting the protein interaction relationships, where lexical item feature, entity distance and keyword are regarded to features.

1) Item feature

In this work, we used the following three types of keyword item features: the keyword items included in the two protein entity names, the keyword items between the two protein entity names, and the keyword items around the two protein entity names.

One protein name may contain multiple words, such as the sentence in Fig. 1, where the bold part indicates a protein entity name, and its characteristic value in the characteristic vector can be denoted as a_1__(IL)-8, a_2__CXCR1, and a_3__CXCR2.

In case that lexical item between two protein entity names is absent, then the characteristics are considered dull. Such as, in the sentence in Fig. 1, the word “and" between protein CXCR1 and protein CXCR2 is expressed as b_1__and in the characteristic value in the characteristic vector.

Given the two proteins, CXCR1 and CXCR2, in the sentence in Fig. 1, the three words at the left side of CXCR1 are “through,” “their” and “receptors” and their characteristic values in the characteristic vector can be expressed as l_1__through, l_2__their, l_3__receptors. Lexical item is absent at the right side of CXCR2, and this feature item is set to dull.

2) Keyword feture

Many words (keywords) around or between two protein entities can designate the protein relationship, including “has” and “receptors”. In this paper, when a keyword emerges around or between two proteins, the keyword is inserted to the keyword form (there are about 600 keywords in the keyword form). As for the sentence in Fig. 1, the corresponding key word, “receptors” are found in the key word form, and its characteristic value in the characteristic vector is expressed as k_receptors.

3) Entity distance entity

The number of interval words between two proteins is called distance. The shorter the distance, the closer the relationship. Therefore, a shorter distance between two proteins demonstrates a higher possibility of their interaction. If the inter-entity distance is equal to or less than three words, then the corresponding characteristic value is expressed as d_3; if the inter-entity distance is greater than three words but equal to or less than eight words, then the corresponding characteristic value is expressed as d_8; if the inter-entity distance is greater than eight words but equal to or less than 15 words, then the corresponding characteristic value is expressed as d_15; if the inter-entity distance is greater than 15 words, then the corresponding characteristic value is expressed as d_16.

The characteristics of two protein entities (IL)-8 and CXCR1 extraction characteristics in the sentence in Fig. 1 are expressed in Table 1.

**Table 1:** (IL)-8 and CXCR1 characteristics

Characteristic name	Characteristic value
Lexical item in the two	a_1__(IL)-8, a_2__CXCR1
Protein names	
Lexical item between the	b_1__has, b_2__an, b_3__important,…
Two protein names	b _1_7_their, b _1_8_receptors
Lexical item around the	l_1__Interleukin, r_1__and
Two protein names	
Key word feature	k_receptors
Entity distance entity	d_16

In this work, we employed the radial-based function as the kernel function for calculating the feature vector (Formula (4)), in which s indicates the covariance matrix.

1 $$\begin{array}{@{}rcl@{}} K(x,y) = \exp \left[ -\frac{|| x - y ||^{2}}{2s^{2}} \right] \end{array} $$

### Extension dependency path tree kernel

Formula (5) is the definition of extension path dependency path tree kernel which is one of convolution tree kernel (“c” which is in the lower right corner is convolution). Formula (5) shows that the tree structure is the representation of the protein entity. And the similarity of semanteme between syntax analysis tree T_1_ and T_2_ is calculated by the same number of structural subtree. Calculation process is as follows: first, the big tree is broken down into many different sub-trees; second, calculating the similarities of these sub-trees; third, the similarity of the big tree is got by summing the similarity of the sub-trees. The dependence path tree kernel [44] and the shortest path tree kernel [45] is two of classical convolution tree.

2 $$\begin{array}{@{}rcl@{}} K_{c}(T_{1},T_{2}) = \sum_{n_{1} \in N_{1}}\sum_{n_{2} \in N_{2}}\Delta\left(n_{1},n_{2}\right)\ \end{array} $$

**Fig. 2:**
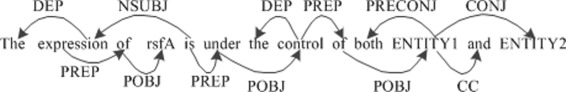
Demonstration of extension dependency path tree kernel



In this article, original dependency path tree kernels are selected for the extension to form the tension dependency path tree kernels. A dependence relationship analysis is conducted (the analysis process is shown in Fig. 2) using “The expression of rsfA is under the control of both ENTITY1 and ENTITY2.” as example. The path tree between ENTITY1 and ENTITY2 is “(DEPENDENCY(CONJ(ENTITY1,ENTITY2))).” Apparently, the information of this tree is insufficient for the judgment of the inter-entity relationship. The solution provided hereby is used to extend the length of the dependency path when the path length is less than three. The path between ENTITY1 and ENTITY2 in the above example can be extended into “(DEPENDENCY(PREP(control, of)) POBJ((of, ENTITY1)) (CONJ(ENTITY1, ENTITY2))).” The algorithm is shown in Algorithm 1.

Where, n_1_ and n_2_ is root node of T_1_ and T_2_; *λ*(0 <*λ*< 1) is the attenuation factor;Nl(n_1_) at line 06 is the number of child nodes of n_1_; n_1_ and n_2_ have the same generative, so Nl(n_1_) = Nl(n_2_); In which cl(n,k) is the k^*th*^ child node of node n; *Δ*(cl(n_1_,k),cl(n_2_,k)) represents calculating the number of same subtrees between tree T_1_ and T_2_ by a recursive algorithm. Hence, the time complexity of algorithm is O(n_1_log(min(n_1_,n_2_))).

The function value between the same trees is much larger than that of different trees when the scale of the tree is very large. We adopted two ways to stop the function value become too much large: a) The function value is normalized by formula(6); b) In order to reducing the impact of subtree scale, we imported the attenuation factor *λ* to multiple the similarity contribution of the subtree on its father node.

3 $$\begin{array}{@{}rcl@{}} K'(T_{1},T_{2}) = \frac{K(T_{1},T_{2})}{\sqrt{K(T_{1},T_{1})K(T_{2},T_{2})}} \end{array} $$

### Tag Graph kernel

#### **Definition 1**

Graph kernel: set G as a finite or infinite graph set, and function *κ* : G ×*G*→R is called one graph kernel. In the presence of one Hilbert space (which is probably infinitely dimensional) F and one mapping *ϕ* : G →F thus, all the points g, g ^′^∈G, *κ*(g,g ^′^)= <*ϕ*(g), *ϕ*(g ^′^) > and <·,·> represents the dot product of Hilbert space F.

The current graph kernel methods are mainly divided into three categories: diffuse graph kernel, volume graph kernel, and path graph kernel. The authors of this article propose the tag graph kernel method. The core is used to compare the quantity of public channels of the two graphs through hashtag to measure their similarity.

#### **Definition 2**

Directed tag graph: given v is one node set, *ε* is one directed edge set and *ε*⊂*ν*×*ν*,*κ* is a tag set, and m ⊂*ν*×*κ* is a mapping from *ν* to *κ*, then graph G = (*ν*,*ε*,m) is a directed tag graph.

#### **Definition 3**

Adjacency matrix: given [E] _*ij*_ = 1 ⇔ (*ν*_*i*_,*ν*_*j*_) ∈*ε*, and [E] _*i*_ *j*≠ 1 ⇔ (*ν*_*i*_,*ν*_*j*_) ∉*ε*, then matrix E is an adjacency matrix of directed tag graph G.

#### **Definition 4**

Tag matrix: given tag set *κ* = { *κ*_1_,*κ*_2_,···}, if [L] _*r*_ *i* = 1 ⇔*κ*_*r*_ = label(*ν*_*i*_), and [L] _*r*_ *i* = 0 ⇔*κ*_*r*_≠ label(*ν*_*i*_), then matrix L is the tag matrix of directed tag graph G.

#### **Definition 5**

Matrix inner product: matrix A and matrix B are the matrices of two m ×n, and the inner product of matrix A and matrix B is defined as 〈A,B 〉 = $\sum \limits _{i=0}^{m}\sum \limits _{j=0}^{n}{\mathrm {A}}_{ij}{\mathrm {B}}_{ij}$.

Given G and G ^′^ as two directed tag graphs, on the basis of hashtag, the all-paths hashtag graph kernel function is shown as Formula (7):

4 $$\begin{array}{@{}rcl@{}} \begin{aligned} K&(G,G')\\ &= \sum\limits_{r=0}^{r'}\beta_{r}\left\langle L{{~}_{r}}\left(\sum\limits_{i=0}^{\infty}\xi^{i}E^{i}\right)L{{~}_{r}^{T}},L'{{~}_{r}}\left(\sum\limits_{i=0}^{\infty}\xi^{i}E^{i}\right)L'{{~}_{r}^{T}} \right\rangle \\ &= \sum\limits_{r=0}^{r'}\sum\limits_{m=0}^{|k|}\sum\limits_{n=0}^{|k|}\beta_{r}\left[L{{~}_{r}}\left(\sum\limits_{i=0}^{\infty}\xi^{i}E^{i}\right)L{{~}_{r}^{T}} \right]_{mn}\left[L'{{~}_{r}}\left(\sum\limits_{i=0}^{\infty}\xi^{i}E^{i}\right)L'{{~}_{r}^{T}} \right]_{mn} \end{aligned} \end{array} $$

where, E and E ^′^ are the adjacency matrices of G and G ^′^, respectively, and L_0_,L _1_,···,L _*r*_, and L0′,L1′,···,L*r*′ are the hashtags of G and G ^′^, respectively. Matrix [E^*n*^]_*i*_ *j* represents the number of all paths in directed tag graph G with a length of n from node *ν*_*i*_ to node $\nu _{j}.\sum \limits _{i=0}^{\infty }\lambda ^{i}{\mathrm {E}}^{i}$ can fuse all paths with different lengths between different nodes into graph G. K is the set consisting of all hashtags, r ^′^ is the upper limit of hashtag top class, and *ξ*(0 <*ξ*< 1) is the path weight parameter of adjacency matrix. *β*_*r*_(*β*_*r*_>0) is the top class of hashtags, and the setting of *β*_0_,*β*_1_,···,*β*_*r*_ can effectively distinguish the effects of the hashtag at different top classes on the different categories of tasks.

### Kernel fusion

The three kernel methods used in this article have their own advantages and disadvantages. The feature-based kernel is simple and effective but cannot obtain the sentence structural information. Extension dependency path can obtain the sentence structural information but ignores the deep grammar information. Tag graph kernels can utilize both the results of the grammar analysis and the characteristics of words but ignores the words with a relatively long distance and the path similarity of over three words. To sum up, the authors of this article propose a method based on the multi-kernel fusion to extract biological entity relationships. For each kernel, the similarity is measured according to its field, as shown in Formula (8).

5 $$\begin{array}{@{}rcl@{}} K(x,y) = \sum_{i=1}^{m}K_{i}(x,y) \end{array} $$

where i represents the quantity of kernels, m=3. To achieve the kernel fusion of different analysis structures, the feature weight *η* is imported, and $\eta _{i}>0,\sum \limits _{i}\eta _{i}= 1$. However, the kernel weighted sum is used to replace the simple multi-kernel summing, as shown in Formula (9):

6 $$\begin{array}{@{}rcl@{}} K(x,y) = \sum_{i=1}^{m}\eta_{i}K_{i}(x,y) \end{array} $$

At this point, the single-kernel target function is turned into as follows:

7 $$\begin{array}{@{}rcl@{}} L_{d} = \sum_{t}\alpha^{t} - \frac{1}{2}\sum_{t}\sum_{s}\alpha^{t}\alpha^{s}r^{t}r^{s}\sum_{i}\eta_{i}K_{i}\left(x^{t},x^{s}\right) \end{array} $$

The multi-kernel combination also appears in Discriminant (11):

8 $$\begin{array}{@{}rcl@{}} g(x) = \sum_{t}\alpha^{t}r^{t}\sum_{i}\eta_{i}K_{i}\left(x^{t},x^{s}\right) \end{array} $$

The value of *η*_*i*_ is used through training, and the value determines the role of the corresponding kernels in the discriminant.

## Results and discussion

To evaluate the multiple-kernel-learning-based method proposed herein, we conducted computational experiments and compared with the existing method.

### Experimental evaluation index

In the biomedical entity relationship extraction research, there are three evaluation indices which are the following: (Precision, P), (Recall, R) and (F-score, F).

9 $$\begin{array}{@{}rcl@{}} P = \frac{TP}{TP+FP} \end{array} $$

10 $$\begin{array}{@{}rcl@{}} R = \frac{TP}{TP+EN} \end{array} $$

11 $$\begin{array}{@{}rcl@{}} F = \frac{2*P*R}{P+R} \end{array} $$

Where TP represents the number of correctly categorized positive examples, TN represents the number of correctly categorized negative examples, FP represents the number of wrongly categorized positive examples, and FN represents the number of wrongly categorized negative examples. P refers to the precision of the algorithm, and R refers to the integrity of reaction algorithm. F value is the harmonic mean of the two evaluation indices of P and R and is currently the main evaluation index for the current biomedical entity relationship extraction study.

### Experimental corpus

In this section, we used five evaluation corpuses [46] which are authoritative evaluation corpuses in the biomedical entity relationship extraction research. Statistical information on the five experimental corpuses, Aimed, IEPA, BioInfer, HPRD50, and LLL, are shown in Table 2.

**Table 2:** Statistical form of corpus information

Corpus set	Number of texts	Number of sentences	Number of positive examples	Number of negative examples	Total number of examples
Aimed	225	1955	1000	4834	5834
IEPA	50	145	335	482	817
BioInfer	863	1100	2534	7132	9666
HPRD50	200	486	163	270	433
LLL	45	77	164	166	330

### Experimental results

All-paths graph kernel method [43] is one of the most typical methods in the protein relationship extraction study. Table 3 shows the comparison of tag graph kernel method and all-paths graph kernel method in terms of their performance in the five corpus sets. Evidently, the performance of the tag graph kernel method in five corpus sets is superior to that of the all-paths graph kernel method. The P, R, and F value of tag graph method in Aimed corpus are 50.82, 69.76, and 58.61%, respectively. The corresponding values of all-paths graph kernel method are 44.97, 65.82, and 55.46%, respectively. The P, R, and F value of tag graph kernel method in other four evaluation corpuses are 2-5% higher than that of all-paths graph kernel. The results indicate that the overallperformance of tag graph kernel method is superior to that of all-paths graph kernel.

**Table 3:** Comparison between tag graph kernel and all-paths graph kernel in terms of their performance

	Tag graph kernel method			All-paths graph kernel		
Corpus set	P	R	F	P	R	F
BioInfer	51.64	68.92	59.73	46.89	62.13	57.25
Aimed	50.82	69.76	58.61	44.97	65.82	55.46
HPRD50	55.64	67.81	70.01	49.76	64.38	68.21
IEPA	61.58	76.91	74.23	56.48	72.36	70.65
LLL	71.92	70.84	77.43	67.19	66.95	72.68

In order to compare two kinds of kernel fusion methods with the three simple kernel methods, we conducted experiments on the BioInfer corpus which is moderate scale. The results are shown in Table 4. In the three separate kernel methods, the tag graph kernel method proposed herein has the best performance followed by the extension dependency path tree kernel. The three kernel methods have a better performance than the single kernel methods. Furthermore, two kernels fuse methods which one is tag graph kernel method obtained the better performance. The P, R and F value of feature kernel and tag graph kernel fuse methods is 53.43, 71.62 and 61.30%, respectively. The P, R and F value of feature kernel and tag graph kernel fuse methods is 55.47, 70.29 and 60.37%, respectively. Experiment results have indicated that the performance of the two kinds of kernel fusion methods is better than that of simple kernel. Hence, the fussed kernel methods indeed improve the performance of protein relationship extraction method.

**Table 4:** Performance of different kernel methods in BioInfer corpus

Method	P	R	F
Characteristics-based kernels	45.61	63.57	56.24
Extension dependency path tree kernel	41.32	69.76	52.58
Tag graph kernel	51.64	68.92	59.73
Feature kernel + path tree kernel	49.86	70.12	60.25
Feature kernel + tag graph kernel	55.43	71.62	61.30
Path tree kernel + tag graph kernel	55.47	70.29	60.37

As shown in Table 5, the three-kernel-fused methods and fused kernel methods remain relatively stable in the five kinds of corpus sets. The fused kernel method has the best performance in all aspects, and the proposed tag graph kernel method has the second best performance. The parameters in the tag graph are the parameters with the best results after r ^′^ and B _*r*_ have gone through a large amount of training. Compared with P and R, the F value in the five corpuses sets changes greatly. For example, the F value of the four methods in the BioInfer corpus ranges from 52 to 62%, whereas the F-value in the LLL corpus ranges from 68 to 91%. Such result is mainly due to the changes in the distribution of positive and negative changes of corpus, which greatly affect the F value, whereas other evaluation parameters are insensitive to the changes in the positive and negative example ratio in corpus. The negative examples in Aimed and Bioinfer corpuses far outnumber the positive examples. Thus, the F value of the two corpuses is significantly lower than that of other corpuses, such as LLL.

**Table 5:** Performance of different kernel methods in five types of corpuses

Corpus set	Evaluation parameters	Characteristics- based kernels	Extension path dependency kernel	Tag graph kernel	Kernels from
					three-kernel fusion
Aimed	P	45.34	42.31	50.82	57.45
	R	61.25	68.54	69.76	72.31
	F	55.36	52.63	58.61	60.98
IEPA	P	56.84	52.48	61.58	73.82
	R	72.92	69.35	76.91	81.06
	F	87.15	63.79	74.23	79.57
BioInfer	P	45.61	41.32	51.64	91.69
	R	63.57	69.76	68.92	71.62
	F	56.24	52.58	59.73	62.35
HPRD	P	50.26	49.96	55.64	61.87
	R	67.59	66.31	67.81	72.35
	F	75.38	69.78	70.01	85.48
LLL	P	53.59	83.34	71.92	75.69
	R	70.12	69.78	70.84	78.37
	F	68.43	88.03	77.43	90.12

## Conclusion

In this paper, a tag graph kernel method used hashtag was proposed, which is combined with extension-path-tree-kernel-based method and characteristic-kernel-based method, a fused kernel learning method was further proposed. Experimental results indicate that the P, R and F value of the tag graph kernel method is higher on five evaluation corpuses in comparison with the all-paths-graph kernel method. And the performance of multi-kernel fusion methods proposed herein is the best of all of methods used in this article. Obviously, multi-kernel fusion methods can make up for the defect in simple kernel and improve the performance of protein relationship extraction method.
